# Factors associated with work ability among primary health care professionals: an integrative review

**DOI:** 10.47626/1679-4435-2022-954

**Published:** 2024-08-05

**Authors:** Marília de Souza Maia, Rodrigo Fernandes Weyll Pimentel, Regina de Souza Moreira, Cristiane Pereira Novaes, Sabrina Batista Steele, Magno Conceição das Merces, Argemiro D’Oliveira-Júnior

**Affiliations:** 1 Programa de Pós-Graduação em Ciências da Saúde, Universidade Federal da Bahia, Salvador, BA, Brazil; 2 Departamento de Ciências da Vida, Universidade do Estado da Bahia, Salvador, BA, Brazil

**Keywords:** work capacity evaluation, health personnel, primary health care, avaliação da capacidade de trabalho, pessoal de saúde, atenção primária à saúde

## Abstract

The self-perception of the individual in relation to their health, work, and lifestyle is considered as work ability, a concept of various dimensions, characterizing a multidimensional and versatile construct, because it encompasses physical, mental, and social prerequisites. This study aims to identify the factors associated with work capacity among primary health care professionals. An integrative review was conducted from March to June 2020, following this eligibility criteria: studies in Portuguese, Spanish, and English; from 1996, available on PubMed, SciELO, LILACS, and Cochrane databases; longitudinal studies, clinical trials, and crosssectional studies; among primary health care professionals; which used the Work Ability Index. Three articles were found, two in English and one in Portuguese. It was concluded that further studies should be conducted, with primary health care professionals, in order to propose measures that can reduce inadequate capacity rates and better understand the variables associated with work ability.

## Abstract (pt)

A autopercepção do indivíduo em relação à saúde, ao trabalho e ao estilo de vida é considerada como a capacidade para o trabalho, um conceito de várias dimensões, caracterizando um constructo multidimensional e versátil por englobar pré-condições físicas, mentais e sociais. Este trabalho teve como objetivo identificar os fatores associados à capacidade para o trabalho entre profissionais de saúde da atenção primária. Foi realizada uma revisão integrativa no período de março a junho de 2020, seguindo os critérios de elegibilidade: artigos em português, espanhol e inglês; trabalhos disponíveis a partir de 1996 nas bases PubMed, SciELO, LILACS e Cochrane; estudos longitudinais, ensaios clínicos e estudos transversais; artigos com profissionais da saúde da atenção primária; e trabalhos que usaram o índice de capacidade para o trabalho. Foram encontrados três artigos, sendo dois em inglês e um em português. Concluiu-se que novos estudos devem ser realizados, com profissionais da equipe da atenção primária, a fim de propor medidas que possam reduzir taxas de capacidade inadequadas e gerar mais conhecimento sobre as variáveis associadas à capacidade para o trabalho.

## INTRODUCTION

The World Health Organization (WHO) has raised concerns about work-related aging process.^1^ The effects of demographic transition on the work process and changes in production relations have sparked interest in studies on work ability (WA).^2^

In this sense, the individual self-perception as to health, work, and lifestyle is characterized as WA, involving a concept with several dimensions and creating a multidimensional and versatile construct as it encompasses physical, mental, and social prerequisites on which no consensus has yet been reached. This creates a level of complexity around this construct.^3,4^

Thus, WA is the result of dynamic processes that occur between the individuals own resources and their work. It is also characterized as how well a worker is now or will be in the near future, and the level of qualification they have to perform their activities in the workplace, given the demands of their health condition and physical and mental capacity,^5-7^ considering a number of factors, such as sociodemographic elements, lifestyle, aging process, and relationships and the representation of work.^6^

The Finnish Institute of Occupational Health (FIOH) designed studies that correspond to the first relevant theoretical framework in this area of knowledge around the 1980s. These studies provided the theoretical basis for important findings on the determinants, consequences, and measures to be taken to intervene and support government policies aimed at maintaining WA, creating the work ability index (WAI), which was translated into Portuguese in 1996.^5^ The WAI is designed for occupational health services, and scientific studies can be conducted due to its accuracy. Its results are reproducible and can be used in investigation and/or follow-up at the individual and collective levels, while also allowing for the assessment of the functional capacity of the worker and associated factors.^3^

It is important to note that many professions, in addition to mental demands, also require physical effort, such as lifting and carrying weights, repetitive and sudden efforts, inappropriate postures, simultaneous stooping, and postural overload. These positions require health promotion measures, considering the possibility of loss of WA, whether in health care or other areas.^6^

Cordeiro & Araújo^3^ emphasize that the professionals targeted in the investigation were mostly nursing professionals (nurses, nursing assistants, and nursing technicians) and workers in the production sector (factories and multinationals producing school and office materials, textiles, food, and drink). Silva et al.^2^ found that the highest prevalence of self-reported illnesses or injuries was attributed to musculoskeletal disorders. As for the diseases that the physician had diagnosed, 25.5% of the workers were classified as hypertensive, 23.5% as obese, 15.3% reported having a mild/severe emotional disorder, and 10.2% had skin allergies.

As a result, maintaining WA correlates with better health and working conditions, both in terms of interpersonal relationships and the environment. In this manner, it is reproduced as a better quality of life both inside and outside the workplace, increased productivity, better use of pensions, and lower costs and expenses in the public health and social security sector.^7^

In view of the above, this study aimed to identify the factors associated with WA among primary health care professionals in the light of the literature.

## METHODS

We conducted an integrative review, which is a resource used to summarize the results found in studies on a topic or question in an organized and comprehensive manner. It is so called because it offers more extensive information on a subject/problem, thus forming a body of knowledge.^8^

The recommendations of the Preferred Reporting Items for Systematic Reviews and Meta-Analyses (PRISMA) checklist were followed. The PICO strategy was used to create the guiding question. This strategy is an acronym for Patient, Intervention, Comparison and Outcomes.^9^ The P referred to health professionals, I to factors influencing WA, C to comparisons between levels of scientific evidence, and O to the possible prevalence of WA. This led to the following guiding question: “What scientific evidence is available in the literature on factors associated with WA among primary health care professionals?”

Publications were searched from March to June 2020, using the Boolean operator AND and truncation techniques. The studies were searched in the following databases: Scientific Electronic Library Online (SciELO), National Library of Medicine National Institutes of Health (PubMed), Cochrane Library and Latin-American and Caribbean Center on Health Sciences Information (LILACS). The keywords were defined according to the Medical Subject Headings (MeSH) and Descritores em Ciências da Saúde (DeCS), in Portuguese, Spanish, and English: Work Capacity Evaluation, Evaluación de Capacidad de Trabajo; Professional de Saúde, Health Personnel, Personal de Salud; and Atenção Primária em Saúde, Primary Health Care, Atención Primaria de Salud.

Eligible articles were: published in Portuguese, Spanish, and English; available electronically on these databases from 1996 (as this is the year the instrument was translated into Brazilian Portuguese); longitudinal studies, clinical trials, and cross-sectional studies; studies with a target population of primary health care professionals; and studies using the WAI to assess WA. The exclusion criteria were: studies unrelated to the subject; that used the WAI incompletely; and instrument validation studies. Duplicates were considered only once.

**Figure 1:**
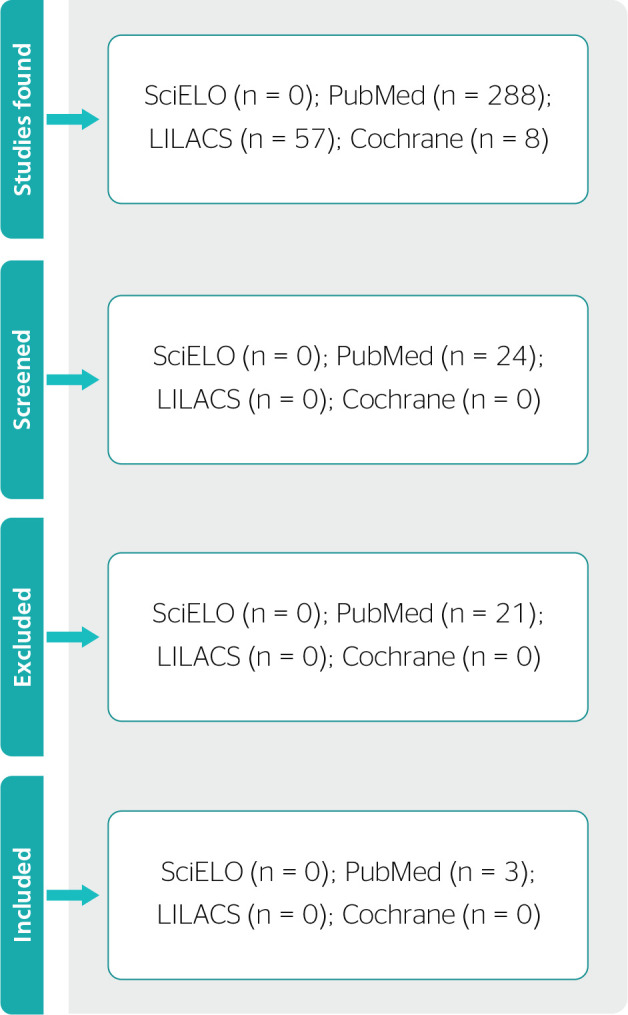
Flowchart of the screening process of the reviewed studies according to database.

The screening method was divided into three phases. The first screening phase was performed through reading the titles of the studies; the second phase involved screening through reading the abstract; and the third phase was completed through reading the study in its entirety. Figure 1 shows a flowchart of the screening process of the reviewed studies.

SciELO = Scientific Electronic Library Online; PubMed = National Library of Medicine National Institutes of Health; LILACS = Latin-American and Caribbean Center on Health Sciences Information.

The studies were critically analyzed using the Agency for Healthcare Research and Quality (AHRQ) classification of levels of scientific evidence, which includes 6 levels: (I) evidence from meta-analyses and systematic reviews; (II) evidence from randomized clinical trials; (III) evidence from nonrandomized clinical trials; (IV) evidence from cohort and casecontrol studies; (V) evidence from systematic reviews of descriptive and qualitative studies; and (VI) evidence based on descriptive or qualitative studies.

After critical analysis, a summary table was drawn up to organize the information extracted from the selected studies with the following items: author/ year of publication, location, objective, study design, factors associated with WA, and AHRQ classification. After reading the selected studies in their entirety, they were organized and analyzed according to the criteria above.

## RESULTS

The search in the databases found 353 articles using the descriptors in Portuguese, Spanish, and English: 288 articles in PubMed, 8 articles in Cochrane, and 57 articles in LILACS. No studies were found in SciELO.

Overall, 24 studies were screened from the 353 found in PubMed. After screening, we excluded those that did not meet the eligibility criteria (21 articles), remaining 3 articles.^4,10,11^

From the eligible articles, 2 were in English^10,11^ and 1 in Portuguese.^4^ As for the years of publication, the studies were published between 2013 and 2018. In terms of country, the articles were from Sweden,^11^ the United States of America,^10^ and Brazil.^4^ The authors of these articles fit into the following professional categories: nurse, psychologist, physician, and mathematician.

According to the AHRQ criteria, 1 study was classified as having level of evidence IV (cohort and case-control),^10^ and the other 2 were classified as having level of evidence VI (evidence based on descriptive or qualitative studies).^4,11^ The articles in this review are summarized in Table 1, sorted according to author/ year of publication, location, objective, study design, factors associated with WA, and AHRQ classification.

**Table 1:** Characterization of the publications included in the integrative review according to year/author, location, objective, study design, factors associated with WA, and AHRQ classification

Author/year	Location	Objective	Study design	Factors associated with WA	AHRQ classification
Hatch et al. (2018)^10^	United States of America	o examine how age and burnout interact in predicting physical and psychological capacity for work	Prospective cohort	The lowest WA, based on the total WAI score at month 12, is associated with greater severity of burnout symptoms, while older age and being a woman were associated with lower burnout.	IV
Cordeiro & Araújo (2018)^4^	Brazil	To assess factors associated with WA in primary health care nursing workers in Bahia, Brazil	Cross-sectional exploratory	Higher prevalence of inadequate WA was observed among workers who did not participate in leisure activities, did not exercise, smoked, drank alcohol, and had a high domestic burden.	VI
Arvidson et al. (2013)^11^	Sweden	To assess the association between exercise and WA, using cross-sectional and prospective analyses	Cross-sectional prospective	With an increased level of exercise, using participants in the sedentary group as a reference, the prevalence of individuals reporting low or moderate WA decreased. Adjustments for age, sex, and BMI showed no appreciable change in the estimates, and the result was still statistically significant.	VI

AHRQ = Agency for Healthcare Research and Quality; WA = work ability; WAI = Work Ability Index; BMI = body mass index.

## DISCUSSION

We believe this study is the first to investigate the factors associated with WA among primary care health professionals in the light of the literature in Brazil. The results of this integrative review contribute to acknowledging the emergence of studies and interventions in primary health care, as no studies addressing all health professionals integrated into primary health care were found, highlighting that incapacity for work among these professionals and its outcomes are harmful conditions for occupational health. Several descriptive studies addressing WA were found in the databases, mainly among nursing professionals, mostly in hospitals, and among professionals from other areas.

In addition, WA cannot be considered only in terms of the characteristics of the worker and the demands of the job. This construct needs to understand context and temporality, known as a system that is created on concrete facts by the worker, their work, and their organization.^12^

The articles found were from different regions, namely Sweden, the United States of America, and Brazil, which shows heterogeneity in the samples and results. In Brazil, no studies were found involving the entire primary health care workforce. This is due to the scarcity of publications and, consequently, the inexistence of investment in preventing the health of these workers. This highlights the importance of studies aimed at WA of primary health care professionals.

In relation to WA-associated factors, studies showed heterogeneity of factors, as they were conducted in different locations, yet with a common objective, to assess WA-associated factors. Hatch et al.^10^ showed that factors are 2-dimensional for WA, divided into physical and psychological components, both of which have an impact on WA and the health of these professionals.

The study conducted in Brazil found a high prevalence of inadequate WA among primary health care nursing workers in relation to having a permanent job, working only 1 shift, having developed an occupational disease, being dissatisfied with their WA, and experiencing high work demands.^4^ This contrasts with the Swedish study, which found that self-reported physical activity during leisure time was positively related to WA.^11^

Therefore, health care should not only be the workers own duty, but also the contracting party, and it is of the utmost importance to provide adequate working conditions, with safety and quality. Studies report that poor or moderate WA leads to an increase in early retirements, and if optimal WA conditions are provided, this will result in healthy workers, meaning less costs for the health system, life satisfaction, and less illness.^11^

We believe it is important to conduct epidemiological studies with robust analyses to better understand, compare, and evaluate the factors associated with WA among primary health care professionals.

## FINAL CONSIDERATIONS

This study evaluated the factors associated with WA among primary health care professionals, showing the social dimension through the 2-dimensional factors that impact on the health of these professionals and on WA. Among the particularities of the work of health professionals are the mental and physical demands that also lead to postural overload, demanding health promotion measures that consider the possibility of loss of WA.

Studies such as this one reinforce the need for constant investigation into this issue, since a closer look at the reality and needs of these professionals will facilitate the development of alternatives that enable physical, social, and mental well-being, thus culminating in improved WA.

It should be pointed out that insufficient investment in workers’ health in Brazil is detrimental to the professional classes and also to investigation, as few publications have been published on this subject, thus giving this study a certain novelty.

We suggest that contractors pay more attention to developing strategies to expand biopsychosocial care for primary care professionals in order to reduce illness, absenteeism, and the conditions that lead to early retirement.

The limitations of this study include the selection mechanism of articles and the low number of studies that met the eligibility criteria, totaling 3 articles, which may limit the scope of this integrative review.
